# Transmission ratio distortion results in asymmetric introgression in Louisiana Iris

**DOI:** 10.1186/1471-2229-10-48

**Published:** 2010-03-18

**Authors:** Shunxue Tang, Rebecca A Okashah, Steven J Knapp, Michael L Arnold, Noland H Martin

**Affiliations:** 1 Center for Applied Genetic Technologies, The University of Georgia, Athens, GA 30602, USA; 2 Dow AgroSciences LLC, Indianapolis, IN 46268, USA; 3 Department of Genetics, The University of Georgia, Athens, GA 30602, USA; 4 Department of Biology, Texas State University - San Marcos, San Marcos, TX 78666, USA

## Abstract

**Background:**

Linkage maps are useful tools for examining both the genetic architecture of quantitative traits and the evolution of reproductive incompatibilities. We describe the generation of two genetic maps using reciprocal interspecific backcross 1 (BC_1_) mapping populations from crosses between *Iris brevicaulis *and *Iris fulva*. These maps were constructed using expressed sequence tag (EST)- derived codominant microsatellite markers. Such a codominant marker system allowed for the ability to link the two reciprocal maps, and compare patterns of transmission ratio distortion observed between the two.

**Results:**

Linkage mapping resulted in markers that coalesced into 21 linkage groups for each of the reciprocal backcross maps, presumably corresponding to the 21 haploid chromosomes of *I. brevicaulis *and *I. fulva*. The composite map was 1190.0-cM long, spanned 81% of the *I. brevicaulis *and *I. fulva *genomes, and had a mean density of 4.5 cM per locus. Transmission ratio distortion (TRD) was observed in 138 (48.5%) loci distributed in 19 of the 21 LGs in BCIB, BCIF, or both BC_1 _mapping populations. Of the distorted markers identified, *I*. *fulva *alleles were detected at consistently higher-than-expected frequencies in both mapping populations.

**Conclusions:**

The observation that *I. fulva *alleles are overrepresented in both mapping populations suggests that *I*. *fulva *alleles are favored to introgress into *I*. *brevicaulis *genetic backgrounds, while *I*. *brevicaulis *alleles would tend to be prevented from introgressing into *I*. *fulva*. These data are consistent with the previously observed patterns of introgression in natural hybrid zones, where *I*. *fulva *alleles have been consistently shown to introgress across species boundaries.

## Background

The Louisiana *Iris *(Iridaceae) species complex has long been recognized as a study system for examining the evolutionary dynamics of natural hybridization and introgression [1]. It is now widely considered a model system for studying plant evolutionary/speciation genetics [2]. Four phenotypically diverse species comprise this complex: *Iris brevicaulis*, *Iris hexagona*, *Iris fulva*, and *Iris nelsonii*. The four species are broadly sympatric throughout the Mississippi River drainage of east-central North America, with the exception of *I. nelsonii*, which is locally endemic to a single parish in Southern Louisiana. When two or more of the Louisiana *Iris *species are *locally *sympatric, hybrid swarms form [e.g. [3,4]], and this natural hybridization has resulted in the introgression of heterospecific DNA into plants that are phenotypically indistinguishable from the parental species [4-7]. Despite introgressive hybridization occurring in each of the three widely-distributed species, these taxa, for the most part, maintain their phenotypic integrity throughout their ranges, largely due to a number of sequentially acting prezygotic and postzygotic reproductive barriers that serve to reduce the probability of interspecific gene flow [for review see [8]]. Thus, this model system provides evolutionary biologists a unique opportunity to examine the reproductive barriers most important in preventing gene flow between hybridizing taxa, and to evaluate the evolutionary consequences when reproductive barriers are incomplete and natural hybridization takes place. Recent analyses of the *I. brevicaulis*/*I. fulva *species pair - using a quantitative qrait locus (QTL) mapping approach - have resolved the genetic architecture for a portion of the factors that limit and/or promote reproductive isolation and introgressive hybridization [9-15].

A study of the genetic architecture of speciation is necessarily a study of the genetics underlying reproductive isolating mechanisms that prevent gene flow between species. *Iris fulva *and *I. brevicaulis *have a number of such reproductive barriers that reduce the chance for interspecific gene flow. First, the two species' ranges reflect ecogeographic isolation [see [16] for an explanation], such that locally-allopatric populations are often encountered [4,5]. Such ecogeographic isolation would result in increased intraspecific mating because a large proportion [but not all, see [17]] of pollinator flight movements occur between closely-spaced flowers [18,19]. This ecogeographic isolation is likely due to the fact that *I. fulva *and *I. brevicaulis *are adapted to divergent microhabitats [20,21]. *Iris fulva *is normally found in intermittently flooded, forested bayous and swamps, while *I. brevicaulis *most often occurs in drier, shaded riparian-typified hardwood forests [3,20]. As suggested by their habitat associations in nature, Martin et al. [9,10] found under experimental conditions that *I. fulva *is more flood-tolerant, while *I. brevicaulis *is a more drought-resistant species. In this regard, when locally sympatric populations are encountered [e.g. [3,4,6,7,22]], the microhabitat associations of the two species would be expected to reduce interspecific pollen transfer.

There are additional, divergent, reproductive components that interact to reduce the chance for interspecific gene flow between *I. fulva *and *I. brevicaulis*. For example, though the two species must overlap in their flowering times to produce the observed natural hybrid zones, *I. fulva *begins flowering, on average, one month earlier than *I. brevicaulis *[3,23]. Furthermore, in experimental plots planted out into nature, no flowering overlap has been recorded between *I. fulva *and *I. brevicaulis *over three separate flowering seasons [[11], Martin et al. unpublished]. Yet, Cruzan and Arnold [23] did indeed record small windows of flowering overlap in naturally-occurring sympatric populations, indicating that this component leads to only partial isolation between these two taxa.

These two *Iris *species display divergent pollination syndromes as well [12], which results in the attraction of different suites of pollinators [13]. *Iris brevicaulis *possesses blue flowers with prominent white and yellow nectar guides, stiff erect sepals and petals, and short anthers, and is primarily bumblebee-pollinated [13,14]. *Iris fulva *has red flowers with reflexed sepals and petals without nectar guides, protruding anthers, and is primarily hummingbird and butterfly-pollinated [13,14,18,19]. These divergent flowering syndromes result in reduced interspecific foraging bouts between the two species [13]. Furthermore, due to the divergent anther positioning in flowers of the two species, pollen from the two species may be transferred from the anthers to different parts of the pollinators' bodies. Such differential placement reduces even further the chance for interspecific pollen transfer (studies currently underway by Martin et al.). Finally, when interspecific pollen transfer occurs, there is also evidence that conspecific pollen precedence reduces the incidence of F_1 _hybrid formation [24].

Due to these strong, sequentially acting prezygotic barriers, the formation of F_1 _hybrids between the two species has been shown to be extremely rare in nature [25]. However, once established as adult plants, F_1 _hybrids reveal extremely high fitness relative to genotypes of the parental species. These hybrids produce over twice as many asexual growth points in nature, flower at 2-3 times the rate and produce significantly more flowers and fruits than either *I. fulva *or *I. brevicaulis *[15]. Thus, despite their rare formation, these extremely fit F_1 _hybrids can and do backcross with the two pure-species plants, resulting in a number of genotypically diverse hybrid populations throughout the broadly-sympatric species ranges. Indeed, naturally occurring hybrid individuals have been confirmed by both phenotypic and molecular markers [20,21,25,26]. Furthermore, population genetic analyses of hybrid zones have revealed a prominent role for assortative mating, conspecific pollen precedence, and selection in determining the ultimate genetic makeup of late-generation hybrid individuals [23], with adaptive introgression potentially contributing during the formation of natural hybrid zones [reviewed by [8,27,28]].

Genetic mapping is a powerful tool to identify the number, location, distribution, effect, and magnitude of the genetic factors underlying species differences, introgressive hybridization, reproductive barriers, and hybrid speciation [e.g. [2,29-36]]. Using two reciprocal BC_1 _mapping populations between *I. fulva *and *I. brevicaulis*, Bouck et al. [14] produced independent BC_1 _linkage maps by scoring segregation patterns of dominant *Iris *retroelement (IRRE) markers. The use of these maps and QTL analyses made possible the determination of the underlying genetic architecture of many of the reproductive barriers described above [habitat isolation: [9,10]; flowering phenology: [11]; pollinator isolation: [13,14]; hybrid fitness: [15]]. These analyses have indicated that a complex genetic architecture underlies most barriers examined. In general, many QTLs contributed to the additive genetic variation observed in backcross hybrids, and these additive QTLs also varied with respect to the direction of their effects (i.e. introgressed *I. fulva *alleles may cause BCIB hybrids to either flower earlier *or *later, depending on which QTL is examined). Epistatic interactions between otherwise additive QTLs commonly contribute to phenotypic variation. In addition, QTLs have been detected that act epistatically (2 × 2 epistasis), yet do not contribute additive effects [15]. In sum, these findings provide support for the notion that the *Iris *genome is potentially a mosaic with respect to gene flow [e.g. see [37,38]], with some regions of the genome being permeable to introgression because the QTLs contained within these regions promote a reduction in reproductive isolation. However, these studies also provide support for the "genic view of speciation" [2,39,40], wherein a small number of genes (or genomic regions), may be sufficient to prevent the complete fusion of hybridizing populations, even in the face of extensive gene flow.

The genetic maps developed by Bouck et al. [14] have been useful tools for examining the underlying genetic architecture of reproductive isolation and introgression between *I. fulva *and *I. brevicaulis *[9-15]. However, because the markers (i.e. *Iris *retroelement- IRRE); [14,41] used to construct the maps were dominantly inherited, there were also some limitations for the QTL analyses. The maps were developed from each of two reciprocal hybrid populations (first-generation backcrosses to *I. brevicaulis *- hereafter referred to as BCIB, and first-generation backcrosses to *I. fulva *- hereafter referred to as BCIF), with dominant *I. fulva *markers segregating in the F_1 _to produce the BCIB map, and dominant *I. brevicaulis *markers segregating in the F_1 _to produce the reciprocal BCIF map. Because of the dominant inheritance patterns, the two maps obtained were unlinked and it is therefore unknown whether or not QTLs detected in each of these separate linkage maps are located on homologous linkage groups. Here, we present two new linkage maps based on expressed sequence tag (EST) - associated microsatellite loci. Given the codominant inheritance of microsatellites, homology of individual markers can be determined, and the two maps developed from the different reciprocal mapping populations can be linked. We report on patterns of transmission ratio distortion (TRD) of these two novel microsatellite maps, and comment as to whether such patterns promote or inhibit introgression of heterospecific alleles. We also note the utility of these new maps for future QTL mapping studies.

## Methods

### Description of Mapping Populations

Two reciprocal interspecific backcross 1 (BC_1_) mapping populations, BCIB and BCIF, were produced from crosses between *I. brevicaulis *genotype IB25 (previously referred to as IB72 by [15,16,19-22,25] and *I. fulva *genotype IF174 [22]. The *I. fulva *individual, IF174, was collected from a wild population in Terrebonne Parish, Louisiana, USA, and the *I. brevicaulis *individual, IB25, was collected from a wild population in St. Martin Parish, Louisiana, USA. Clones from the same individuals (IF174 and IB25) were utilized to make the initial F_1 _parents of the backcross populations, using IB25 as the seed parent and IF174 as the pollen parent. Two different F_1 _individuals, designated as F_1_(2) and F_1_(3), were used as pollen parents to produce multiple BC_1 _hybrids. Separate F_1 _hybrids were used as pollen parents because flowering had ceased in the F_1_(2) parent prior to the initiation of *I. brevicaulis *flowering. The F_1_(2) plant was thus utilized to pollinate flowers from several clones of IF174, while the F_1_(3) plant was utilized to pollinate several flowers from a number of clones of IB25. Ultimately, several hundred seeds were generated for each reciprocal backcross mapping population. These BC_1 _hybrid seeds were planted in the greenhouse at the University of Georgia in 1999 and monitored for germination success. Successfully-germinated seeds were transplanted into six-inch azalea pots shortly after germination, and plants have been repotted annually from a single rhizome. The current BCIB population housed at the University of Georgia has 230 BC_1 _plants, while BCIF consists of 180 BC_1 _plants. Additional genotypes are located in field plots in Louisiana [described in [9-11,13,15]] as well as at Texas State University - San Marcos. A subset of 94 BCIB and 92 BCIF BC_1 _hybrids from the University of Georgia collection were used in the genetic map construction described herein. From these individuals, genomic DNA was isolated from leaves using a modified CTAB (cetyltrimethylammonium bromide) extraction method.

### EST-SSR Marker Genotyping

Microsatellite marker development and genotyping was essentially the same as described by Tang et al. [42,43] and Tang and Knapp [44]. A total of 1,447 microsatellites were identified from the EST database of *I. brevicaulis *and *I. fulva *at repeat number *n *≥ 5, and 526 EST-microsatellite markers were developed [45]. These 526 markers were screened for utility, functionality, and length polymorphisms in the two mapping parents, IB25 and IF174. To facilitate multiplex genotyping, the expected lengths of the target amplicons were uniformly distributed in the 100 to 450 bp range, and the forward primers were labeled with one of the three fluorophores 6FAM, HEX, and TAMRA. PCR was performed by using 12 μL of reaction mixture containing 1.0 × PCR buffer, 2.5 mM Mg^++^, 0.2 mM each of the dNTPs, 5.0 pmol of each primer, 0.5 units of *Taq *polymerase, and 10 to 15 ng of genomic DNA. 'Touchdown' PCR [46] was used to reduce spurious amplification. The initial denaturation step was performed at 94°C for 1 min, followed by 1 cycle of 94°C for 25 s, 64°C for 25 s, and 72°C for 45 s. The annealing temperature was decreased 1°C per cycle in subsequent cycles until reaching 58°C. Products were subsequently amplified for 33 cycles at 94°C for 20 s, 58°C for 20 s, and 72°C for 45 s with a final extension at 72°C for 20 min.

Amplicon multiplexing was possible because fluorescence labels and allele sizes differed amongst the multiplexed microsatellite markers. Because no allele size information was available for the new microsatellites, we initially multiplexed only three SSR markers (each marker with different fluorescence labels) for parental genotyping. For mapping population genotyping, we were able to multiplex a minimum of eight markers of varying lengths and fluorescence labeling. Each PCR product was diluted 60-100 fold with distilled H_2_O, and pooled. Samples were prepared for genotyping by combining 0.7 to 1.0 uL of the diluted amplicons with 8 uL diluted GeneScan ROX500, the internal-lane size standard. The diluted ROX500 size standard was prepared by mixing 2 uL of original ROX500 size standard (Applied Biosystems, Foster City, Calif., USA) with 100 uL of 100% Formamide. Samples were heated to 92°C for 5 min, chilled on ice for 5 min, and loaded into an ABI 3700 XL Capillary Sequencer (Applied Biosystems, Foster City, CA) for GeneScan. GeneScan Filter Set D was used for data collection; the emission colors of 6FAM, HEX, TAMRA, and ROX were blue, green, yellow, and red, respectively. SSR allele lengths were scored using GeneMapper (Applied Biosystems, Foster City, CA) or Mapmarker (SoftGenetics LLC, State College, PA).

### Genetic Mapping

The genetic maps were constructed using 94 BCIB and 92 BCIF BC_1 _hybrids. Chi-square tests for segregation distortion were performed for each EST-SSR marker using log-likelihood ratio statistics (*G*) of G-MENDEL 3.0 [47]. Genetic maps were constructed using Mapmaker 3.0 [48,49]. The framework maps were constructed at a likelihood odds (LOD) threshold of 7.0 and a maximum recombination frequency threshold of 0.4. Then, we incorporated the unlinked marker loci to the framework maps at LOD scores 5.0 and 3.0. Using the group information from both BCIB and BCIF populations, we assembled 283 of the 285 EST-SSR marker loci into 21 linkage groups (LGs) at LOD threshold ≥ 3.0. Map distances (cM) were calculated using the Kosambi [50] mapping function. For the composite map, the raw genotyping data from both BCIB and BCIF populations was combined for map construction. Of the 285 EST-SSR marker loci genotyped, 222 were genotyped in both BCIB and BCIF, and 63 were genotyped in only one of the BCIB or BCIF populations. For the EST-SSR marker loci mapped in only one population, we used missing data for all of the BC_1 _hybrids from the other population in the composite map construction.

The inferred genome length was estimated by *L *+ (2*tL*)/*n*, as proposed by Fishman et al. [33],

and as proposed by Chakravarti et al. [51], where *L *is the observed length of the genetic map (cM), *n *= *k *- *t *is the number of marker loci intervals, *k*_*i *_is the number of the framework marker loci on the *i*th linkage group, and *i *= 1, 2, ..., *t*, (*t *= 21). The proportion of the genome within *d *cM of a marker locus, assuming a random distribution of framework marker loci, was estimated by 1 - e^-2*dk*/*L *^[51].

**Figure 1:**
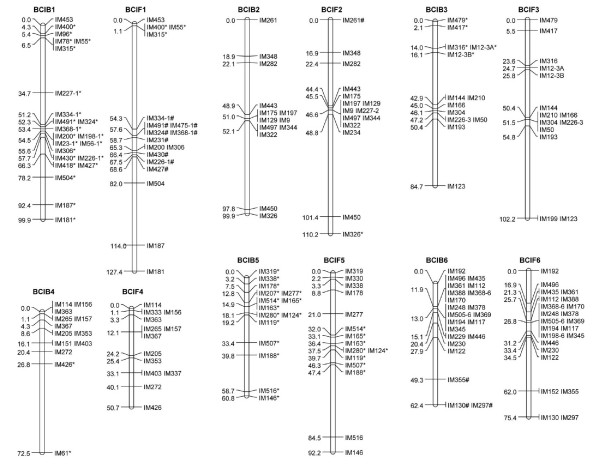
**Transcript genetic linkage maps of *I. brevicaulis *and *I. fulva *based on 283 EST-SSR marker loci genotyped in 94 progeny from the backcross mapping population BCIB, and 92 progeny from the backcross mapping population BCIF**. BCIB and BCIF were reciprocal backcross mapping populations derived from crosses between *I. brevicaulis *(IB25) × *I. fulva *(IF174). The genetic linkage groups were named from 1 to 21 (here groups 1-6) in the order of their genetic map lengths in the composite map (Additional File 1: Supplemental Figure 1). Marker loci showing significant segregation ratio distortion (α ≤ 0.05) in the mapping populations were highlighted with * (overrepresentation of the IF174 alleles) and # (overrepresentation of IB25 alleles).

**Figure 2:**
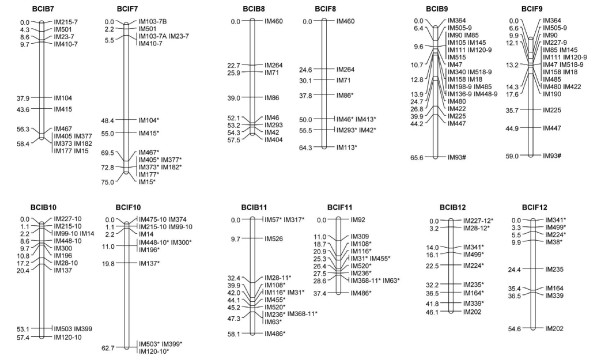
**Linkage groups 7-12. See Figure 1 for details**.

**Figure 3:**
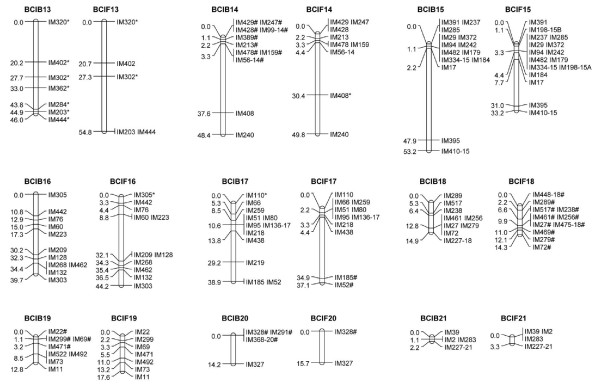
**Linkage groups 13-21. See Figure 1 for details**.

The linkage groups were designated from one to 21 according to the LG lengths in the composite map (Additional File 1: Supplemental Figure S1). A common prefix 'IM' ('*Iris *microsatellite') was used in naming the microsatellite markers. LG number suffixes were used to identify individual loci produced by multilocus markers, e.g., IM56-1 and IM56-14 are loci on LGs 1 and 14, respectively, amplified by the EST-SSR marker IM56. If duplicated loci were mapped to the same LG, then consecutive letters (A, B, C etc.) were used to identify individual loci within the LG, e.g., IM103-7A and IM103-7B are duplicated loci amplified by the IM103 primer pair and mapped at different positions on LG 7 (Figures 1, 2 and 3).

## Results

### EST Microsatellite Marker Genotyping and Polymorphisms

The 526 EST microsatellite markers were screened for utility, functionality, and length polymorphisms in two mapping parents, IB25 and IF174. Of the 526 primer pairs, 399 (76%) amplified distinct bands in at least one of the parents. Of the 399 functional markers, 72 spanned introns larger than 200 bp, and amplified bands larger than 700 bp, which exceed the size range of the ABI 3700 XL Capillary Sequencer; allele sizes of these markers could not be determined and scored (Additional File 2: Supplemental Table S1). The parental individuals IB25 and IF174 were highly heterozygous, indicating that both individuals are members of outcrossing lineages. Of the 327 SSR markers with alleles scored, 213 (65.1%) were heterozygous in IB25, and 163 (49.8%) were heterozygous in IF174. Further, 275 (84%) were polymorphic between IB25 and IF174 (Additional File 2: Supplemental Table S1), and these markers were useful for the current mapping study. We selected 261 of the polymorphic markers and screened them in the two F_1 _hybrids, F_1_(2) and F_1_(3). Some of the polymorphic markers could not be used in genetic mapping because the markers amplified alleles shared by the two parents in addition to the polymorphic alleles, and both parents transferred the shared alleles to the F_1 _hybrids. For example, IM58 amplified 189- and 199-bp alleles in IB25, and 180- and 189-bp alleles in IF174; both parents transferred its 189-bp allele to the F_1_(2) and F_1_(3) hybrids (with homozygous 189-bp alleles), and it is therefore not possible to identify which parents contributed the 189-bp allele in BC_1 _hybrids (Additional File 2: Supplemental Table S1). We found that 24 and 29 polymorphic markers were rendered noninformative in the F_1_(2) and F_1_(3) hybrids, respectively for this reason. Thus, 237 markers in all were genotyped in the BCIB population, and 232 markers were genotyped in the BCIF population (253 different polymorphic markers in all).

### Genetic Maps

Several mapping iterations were performed to produce the final map presented here. At LOD threshold of 7.0 and a maximum recombination frequency threshold of 0.4, the microsatellite markers were assembled into 29, 35 and 26 groups in BCIB, BCIF and composite populations, respectively. At a reduced LOD score of 5.0 (and 3.0), the EST-SSR markers were assembled into 25 (22 at LOD 3.0), 29 (26 at LOD 3.0) and 23 (20 at LOD 3.0) groups in BCIB, BCIF and composite populations, respectively. When the LOD threshold was dropped to 2.5, the marker loci were assembled into 22 LGs in BCIB, 22 LGs in BCIF, and 20 LGs in the composite map. In two cases, the EST-SSR markers from one group in one population were separated into two groups in the other population at LOD threshold ≥ 3.0. Using the group information from both BCIB and BCIF populations, we assembled 283 of the 285 EST-SSR marker loci into 21 linkage groups (LGs) at LOD threshold ≥ 3.0.

A total of 237 polymorphic EST-SSR markers were genotyped in 94 BCIB BC_1 _hybrids, which produced 258 usable marker loci. With the exception of a single locus IM37, all loci coalesced into 21 LGs, presumably corresponding to the 21 haploid chromosomes in both *I. brevicaulis *and *I. fulva *(Figures 1, 2 and 3; Table 1). The map was 1093.6-cM long. The LGs ranged from 2.2 (LG 21) to 99.9 cM (LGs 1 and 2) in length, and had four (LGs 20 and 21) to 23 (LGs 1 and 9) marker loci. The marker densities ranged from 0.7 cM/locus in LG 21 to 8.3 cM/locus in LG 2 with a mean of 4.6 cM/locus for the entire map. Gaps larger than 30.0 cM were observed in LG 2 (45.7 cM), LG 3 (34.3 cM), LG 4 (45.7 cM), LG 10 (32.7 cM), LG 14 (34.3 cM), and LG 15 (45.7 cM) (Figures 1, 2 and 3; Table 1). The inferred total map length ranged from 1414.8 cM [33] to 1419.6 cM [51]; the BCIB map covered 77% of the Louisiana *Iris *genome. Based on this map, 25.9% of the genome is within 1.0 cM and 95.0% of the genome is within 10.0 cM of a SSR marker locus in the BCIB map.

**Table 1:** Number of marker loci, map length, and map density of each linkage group in the BCIB, BCIF and composite genetic maps.

Linkage Group	Number of Marker Loci			Length (cM)			Density (cM/locus)		
	**BCIB**	**BCIF**	**Composite**	**BCIB**	**BCIF**	**Composite**	**BCIB**	**BCIF**	**Composite**
1	23	18	25	99.9	127.4	123.4	4.5	7.5	5.1
2	13	15	15	99.9	110.2	105.2	8.3	7.9	7.5
3	13	14	14	84.7	102.2	92.5	7.1	7.9	7.1
4	13	13	15	72.5	50.7	84.0	6.0	4.2	6.0
5	15	15	16	60.8	92.2	75.5	4.3	6.6	5.0
6	22	23	24	62.4	75.4	68.6	3.0	3.4	3.0
7	13	14	15	58.4	75.0	67.0	4.9	5.8	4.8
8	8	9	10	57.5	64.3	63.6	8.2	8.0	7.1
9	23	19	25	65.6	59.0	62.4	3.0	3.3	2.6
10	12	12	14	57.4	62.7	60.0	5.2	5.7	4.6
11	13	11	15	58.1	37.4	60.0	4.8	3.7	4.3
12	9	8	10	46.1	54.6	57.2	5.8	7.8	6.4
13	7	5	7	46.0	54.8	50.4	7.7	13.7	8.4
14	11	9	11	48.4	49.8	48.7	4.8	6.2	4.9
15	14	16	16	53.2	33.2	41.8	4.1	2.2	2.8
16	11	11	11	39.7	44.2	41.6	4.0	4.4	4.2
17	12	11	12	38.9	37.1	38.7	3.5	3.7	3.5
18	9	11	12	14.9	14.3	16.7	1.9	1.4	1.5
19	8	7	8	12.8	17.6	15.1	1.8	2.9	2.2
20	4	2	4	14.2	15.7	14.9	4.7	15.7	5.0
21	4	4	4	2.2	3.3	2.7	0.7	1.1	0.9
Whole Map	257	247	283	1093.6	1181.1	1190.0	4.6	5.2	4.5

A total of 232 polymorphic EST-SSR markers were genotyped in 92 BCIF BC_1 _hybrids, which produced 249 usable marker loci. Except for IM37 and IM518U, all loci coalesced into 21 LGs, again presumably corresponding to the 21 haploid chromosomes in *I. brevicaulis *and *I. fulva *(Figures 1, 2 and 3; Table 1). The map was 1181.1-cM long. The LGs ranged from 3.3 (LG 21) to 127.4 cM (LG 1) in length, and marker numbers ranged from two (LG 20) to 23 (LG 6). The marker densities ranged from 1.1 cM/locus in LG 21 to 13.7 cM/locus in LG 13 with a mean of 5.2 cM/locus for the entire map. Gaps larger than 30.0 cM were observed in LG 1(53.2 cM), LG 2 (52.6 cM), LG 3 (47.4 cM), LG 5 (37.1 cM), LG 7 (42.9 cM), LG 10 (42.9 cM), and LG 17 (30.5 cM) (Figures 1, 2 and 3; Table 1). The inferred total map length ranged from 1535.4 cM [33] to 1549.0 cM [51]; the BCIF map covered 77% of the Louisiana *Iris *genome. Based on this map, 23.9% of the genome is within 1.0 cM and 93.5% of the genome is within 10.0 cM of a SSR marker locus in the BCIF map.

A total of 285 marker loci from 253 EST-SSR markers were genotyped in BCIB, BCIF, or both populations; 222 marker loci were genotyped in both BCIB and BCIF, and 63 were genotyped in only one of the BCIB or BCIF populations. The marker order was roughly the same in the reciprocal mapping populations. For the purposes of graphical display, we combined the raw genotyping data from both BCIB and BCIF populations, and constructed a composite genetic map (Additional File 1: Supplemental Figure S1, Figure S2). Of the 285 marker loci genotyped, 283 marker loci coalesced into 21 LGs (Additional File 1: Supplemental Figure S1). This composite map was 1190.0-cM long. The LGs ranged from 2.7 (LG 21) to 123.4 cM (LG 1) in length, and had four (LGs 20 and 21) to 25 (LGs 1 and 9) marker loci. The marker densities ranged from 0.9 cM/locus in LG 21 to 8.4 cM/locus in LG 13 with a mean of 4.5 cM/locus for the entire map. Gaps larger than 30.0 cM were observed in LG 1(39.9 cM), LG 2 (48.4 cM), LG 3 (40.2 cM), LG 4 (45.5 cM), LG 7 (34.8 cM), LG 10 (37.4 cM), and LG 15 (33.2 cM) (Additional File 1: Supplemental Figure S1; Table 1). The inferred total map length ranged from 1558.7 cM [33] to 1564.1 cM [51]; the composite map spanned 81% of the Louisiana *Iris *genome. Based on this map, 29.4% of the genome is within 1.0 cM and 96.9% of the genome is within 10.0 cM of a SSR marker locus in the composite map.

### Transmission Ratio Distortion

**Figure 4:**
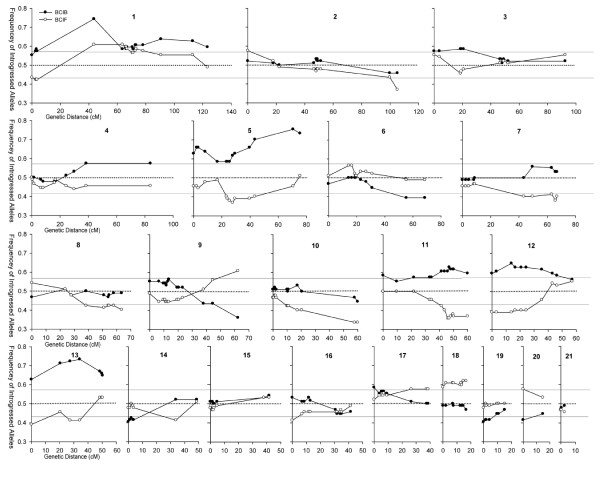
**The observed frequencies of introgressed heterospecific alleles transmitted from F_1 _(2) or F_1 _(3) hybrids to backcross progeny in BCIB or BCIF populations**. The X-axis indicates the genetic distances (cM) of the LGs in the composite map; the Y-axis indicates the transmission ratio of introgressed heterospecific alleles, the IF174 alleles in the BCIB population and the IB25 alleles in the BCIF population. Frequencies > 0.50 indicate an overrepresentation of heterospecific alleles. Frequencies < 0.50 indicate an overrepresentation of homospecific alleles. The expected frequency is 0.50. Data points above and below the stippled lines indicate significant deviations from 0.50 (α = 0.05).

Approximately one-third of the markers in each linkage map revealed significant transmission ratio distortion (TRD - α < 0.05). In the BCIB map, 92 (35.8%) of the 257 mapped marker loci showed significant TRD, while 76 (30.8%) of the 247 mapped marker loci showed significant TRD in the BCIF map (Fig. 4). In both linkage maps, TRD revealed directional bias, with *I. fulva *alleles being significantly overrepresented. In the BCIB map, 79.1% (72/92) of the distorted markers revealed a significant overrepresentation of introgressed *I. fulva *alleles (χ^2 ^= 30.87, d.f. = 1, p < 0.001). In the BCIF map, 67.1% (51/76) of the distorted markers also revealed significant overrepresentation of recurrent *I. fulva *alleles (χ^2 ^= 8.89, d.f. = 1, p = 0.003), at the expense of introgressed *I. brevicaulis *alleles. Significant transmission ratio distortion was thus observed in 138 loci distributed across 19 of the 21 linkage groups in BCIB, BCIF, or both mapping populations (Figure 4).

A visual inspection of the patterns of segregation distortion reveals several regions of "clustering" of distorted markers (i.e. one or more adjacent markers showing significant transmission ratio distortion, Figure 4). The most striking pattern observed was one in which *I. fulva *alleles were significantly overrepresented across *both *mapping populations (i.e. LG 1: 2.7-3.7 cM, LG 5: 23.4-43.5 cM, LG 11: 41.5-60 cM, LG 12: 0-24.4 cM, LG 13: 0, 27.4-34.2 cM). *Iris fulva *alleles were also found to be significantly overrepresented in only one of the two mapping populations for a number of linkage groups (i.e. *BCIB*: LG 1: 90.7-123.4 cM, LG 3: 0-20.8 cM, LG 4: 38.5-84.0 cM, LG 5: 0-16.9, 70.6-75.5 cM, LG 11: 0.0, 21.9-34.4 cM, LG 12: 35.5-46.3 cM, LG 13: 20.4, 48.5-50.4 cM, LG 17: 0 cM, *BCIF*: LG 2: 105.2 cM, LG 7: 43.5-67.0 cM, LG 8: 38.4-63.6 cM, LG 10: 10.0-60.0 cM, LG 14: 33.8 cM, LG 16: 0.0 cM). In contrast, clusters of *I. brevicaulis *alleles were significantly overrepresented in both mapping populations in only two instances (i.e. LG 9: 62.4 cM, LG 20: 0 cM), and overrepresented in only one of the mapping populations in relatively few instances (i.e. *BCIB*: LG 6: 55.4-68.6 cM, LG 14: 0.0-3.8 cM, LG 19: 0.0-4.3 cM, *BCIF*: LG 17: 26.6-38.7, LG 18: 0.0-16.7 cM). Only a single region of segregation distortion was discovered in which heterospecific alleles were overrepresented in *both *mapping populations (LG 1: 63.6-78.1 cM). Strikingly, no regions of segregation distortion were found in which homospecific alleles were significantly overrepresented in *both *mapping populations (Figure 4).

## Discussion

### The Transcript Genetic Maps for Iris

We constructed the first sequence-based genetic maps for *Iris *using codominant (i.e. EST- microsatellite; [45]) markers. Our map construction was based on the same two reciprocal interspecific BC_1 _populations from crosses between *I. brevicaulis *and *I. fulva *utilized to generate the dominant IRRE-based maps described by Bouck et al. [14]. Because of the codominant nature of microsatellite markers, the current maps allow the identification of homologous linkage groups from *I. fulva *and *I. brevicaulis*. Thus, it will be possible to determine whether or not QTLs identified in one mapping population likewise influence quantitative traits in the reciprocal mapping population.

In the current map, more than 80% of the EST- microsatellite markers were polymorphic between IB25 and IF174 and were subsequently mapped in one or both of the mapping populations. The maps consisted of 283 marker loci distributed across 21 LGs, which corresponds to the number of chromosomes identified through karyotyping of these species [52]. The combined map had a length of 1190.0 cM, spanning 81% of the *I. brevicaulis *and *I. fulva *genome, and calculations of map length and map coverage were similar across both non-integrated maps. Based on the shared marker loci, the homology of the LGs in BCIB and BCIF genetic maps were well-established. The marker order was nearly identical in both reciprocal maps, and little evidence of potential genomic rearrangements was found.

The EST- microsatellite loci were not evenly distributed in either of the linkage maps. Substantial clustering was observed in most of the LGs, with complete co-segregation of some markers being observed in almost all of the 21 LGs, even though all the markers were developed from non-redundant unigenes or uniscripts (Figures 1, 2 and 3; Additional File 2: Supplemental Table S1). Significant marker clustering in the present map may be due to a non-random distribution of genes in the *Iris *genome. Since the EST-microsatellite loci are gene-based, the clustering of markers might thus reflect gene-rich regions. Another cause of such non-random distributions of markers could be reduced recombination. For example, centromeric regions of the genome usually reveal suppressed recombination [53-56], and regions of high marker clustering could be associated with such regions.

Only 18 of the 253 polymorphic EST-microsatellite markers produced multiple (2-7) marker loci in the mapping populations (Figures 1, 2 and 3; Additional File 2: Supplemental Table S1); this indicated that the vast majority of the markers were highly conserved throughout the *Iris *genome, and are thus excellent resources for comparative mapping. These 18 multi-locus markers all together resulted in 50 mapped loci in the two maps; 8, 7, 7 and 5 marker loci clustered on the LGs 1, 9, 10 and 7, respectively. We found no apparent syntenic linkage blocks of duplicated EST-SSR marker loci although LG 6 and LG 9, and LG 10 and LG 18 had linkage blocks with two duplicated loci shared (Figures 1, 2 and 3; Additional File 1: Supplemental Figure S1). BLAST indicated that the sequences of these EST-SSR marker belonged to the same gene or pseudogene families.

### Implications of Transmission Ratio Distortion

Approximately 1/3 of all microsatellite markers were significantly distorted in each of the reciprocal backcross maps. This level of distortion is commonly observed in interspecific crosses [14,33,57,58]. Since markers distributed across a linkage group are, by definition, not independent observations, the distorted markers were often found to be clustered in specific regions (Figure 4). These regions of transmission ratio distortion reveal a bias towards *I. fulva*, in that *I. fulva *alleles are largely overrepresented at the expense of *I. brevicaulis *alleles. For instance, in the BCIB mapping population, 18 separate regions were identified in which introgressed *I. fulva *alleles were significantly favored, while in the BCIF mapping population, recurrent *I. fulva *alleles were significantly favored in 12 genomic regions (see results and Figure 4). In contrast, *I. brevicaulis *alleles were significantly overrepresented in only five locations in the BCIB mapping population, and only five locations in the BCIF mapping population. This transmission ratio bias towards *I. fulva *alleles was significant or nearly so in both mapping populations (BCIB: **χ**^2 ^= 7.35, P = 0.007, d.f. = 1; BCIF: **χ**^2 ^= 7.35, P = 0.089, d.f. = 1). Thus, it appears that some causal factor(s) underlie this effect. Whatever the mechanism(s) involved, given that these two species hybridize in nature, this asymmetry in gene flow could have important implications for introgressive hybridization. Namely, we would expect that for a majority of the regions revealing transmission ratio distortion, *I. fulva *alleles might be favored to introgress into a predominately *I. brevicaulis *species-background, while the introgression of *I. brevicaulis *alleles into *I. fulva *would be retarded. Consistent with this prediction, asymmetrical isolation has been observed in natural hybrid zones between *I. brevicaulis *and *I. fulva*, with *I. fulva, I. fulva*-like hybrids, *I. brevicaulis *and *I. brevicaulis*-like hybrids all revealing extraordinarily high probabilities of being sired by *I. fulva*-like genotypes [23].

A number of biological processes may result in transmission ratio distortion in mapping populations. Due to the nature of our crossing design, in which F_1 _hybrids were backcrossed to their original parents, inbreeding depression could cause some instances of transmission ratio distortion. Both original parents were wild-collected, presumably outcrossed, individuals. The high levels of heterozygosity observed in the present analysis and in previous studies [14] corroborate this conclusion. Both parents could thus be carrying lethal or semi-lethal recessive alleles in a heterozygous state. In order for inbreeding depression to manifest as significant transmission ratio distortion, a deleterious allele from the recurrent parent must first be passed on to the F_1 _parent. Then, in producing a backcross individual, the F_1 _must pass on that allele to the offspring, and the recurrent parent must again provide the deleterious allele as well. It is an increase in these homozygous semi-lethal/lethal recessive homozygotes in a mapping population that can ultimately result in transmission ratio distortion. However, such inbreeding depression will only result in introgressed heterospecific alleles being overrepresented, and cannot explain the overrepresentation of recurrent homospecific alleles. In the BCIB mapping population, introgressed *I. fulva *alleles tend to be favored, suggesting that inbreeding depression could play a causal role in much of the observed transmission ratio distortion patterns in this mapping population. However, an examination of transmission ratio distortion patterns in the reciprocal BCIF mapping population indicates that this is likely not the case for many of the distorted regions identified. Were inbreeding depression causing overrepresentation of *I. fulva *alleles in the BCIB mapping population, the same mechanism would not cause such distortion in the BCIF mapping population. However, for six significantly distorted regions in the BCIB map (located on LGs 1, 5, 11, 12, and 13; see results and figure 4), *I. fulva *alleles were also significantly overrepresented in the reciprocal BCIF map. This suggests that some *I. fulva *alleles are selectively favored independent of the genetic background. In contrast, *I. fulva *alleles were overrepresented on LG 1 in the BCIB map, but underrepresented in the BCIF map (**LG 1: **63.6-78.1 cM, Figure 4). This suggests selection for hybridity in this region. Neither of these patterns, where regions of transmission ratio distortion are corellated across both reciprocal maps, are consistent with the expected effects from inbreeding depression.

In other mapping studies, negative interactions between heterospecific nuclear genes have been implicated as the primary causal factor of transmission ratio distortion [14,33,59,60]. Interestingly, the present study reveals little evidence supporting this hypothesis. Not a single instance was observed in which introgressed alleles were underrepresented in *both *populations, indicating that "hybridity" was not universally disfavored across different genetic backgrounds. This may be due largely to the fact that genes conferring postzygotic isolation act mostly in a recessive fashion [reviewed by [61,62]], and loci that could potentially confer hybrid inviability are masked by the recurrent parent's alleles in backcross mapping populations. Cytonuclear incompatibilities can also cause transmission ratio distortion if introgressed nuclear alleles are incompatible with the cytoplasmic genome. Since the original F1 parent contains an I. brevicaulis cytoplasm, any cytonuclear incompatibilities that manifest as transmission ratio distortion should result in an under-representation of I. fulva alleles. Since the opposite was generally observed in this study (and in both maps), are likely not the primary cause of transmission ratio distortion, though in some cases they cannot be ruled out.

In fact, it seems likely that the general overrepresentation of *I. fulva *alleles in both mapping populations is likely not due to inbreeding depression or uniform selection against hybrid genotypes. Rather, a large number of *I. fulva *alleles appear to be selected for over the *I. brevicaulis *allelic counterparts, regardless of the genetic background. This overrepresentation of *I. fulva *alleles could result from a number of factors, including 1) meiotic anomalies in the F_1_, 2) inviabilities of certain gametophytes, 3) pollen competition among the F_1 _hybrid (i.e. the pollen parent in these crosses) gametes, leading to differential fertilization success, 4) differential survival among the resultant backcross hybrid zygotes, 5) differential germination success of the backcross hybrid seeds, and/or 6) differential long-term survival among the resultant hybrid plants.

All of the above factors may play at least some role in promoting segregation ratio distortion. For example, there is evidence suggesting that competition among the F_1 _pollen grains and differential fertilization success together play the most important role in causing the observed overrepresentation of *I. fulva *alleles. *Iris fulva *pollen is much more successful at producing F_1 _seeds than either *I. brevicaulis *or the related *Iris hexagona *[23,24,63-65]. This is partially attributed to the fact that *I. fulva *pollen tubes travel at much faster rates than either *I. brevicaulis *or *I. hexagona *pollen tubes [24,63,64]. Furthermore, *I. fulva *acts as a very restrictive seed parent, such that when reciprocal, competitive crosses are compared, significantly fewer F_1 _progeny are formed in *I. fulva *fruits relative to both *I. brevicaulis *fruits [23,24] and *I. hexagona *fruits [63-66]. Thus, both pollen tube competition and the increased selectivity of conspecific pollen by *I. fulva *have been implicated in promoting asymmetric isolation, which may manifest as segregation ratio distortion favoring *I. fulva *alleles. Likewise, Cruzan and Arnold [23] also detected differential seed abortion which contributed to this same pattern of asymmetric introgression from *I. fulva *into *I. brevicaulis *in a natural hybrid zone.

Since plants were genotyped nine years after they were initially planted in the greenhouse, differential survival among the resultant "adult" hybrid plants could have contributed to the transmission ratio distortion as well. Indeed, Martin et al. [9], using the same mapping population as the current study, found significant differences between the BCIB and BCIF hybrids in survivorship rates after six years under greenhouse conditions. The mortality rate of all BCIF hybrids (25.1%) was roughly twice that of BCIB hybrids (12.3%). Thus, this "adult mortality" could result in significant transmission ratio distortion, and may in fact be responsible for some of the distorted regions in the present study. However, it is quite clear that *I. fulva *genotypes were largely selected *against *[9], which cannot account for the fact that *I. fulva *alleles were generally found to be favored in the present study.

## Conclusions

Transmission ratio distortion in plants can be caused by any number of post-pollination factors that favor certain hybrid genotypes that act prior to the point at which the mapping populations are assayed. Since reproductive barriers act in a sequential order, early-acting barriers (such as those that cause transmission ratio distortion) have the potential to be more effective at restricting gene flow than later acting barriers, even if the absolute strength of the barriers is the same [8,61,67-69]. As already mentioned, natural Louisiana *Iris *hybrid zones reveal strong asymmetries with respect to gene flow, with *I. fulva *alleles being much more likely to introgress into *I. brevicaulis *than the reverse [23]. All of our experimental data, the present data set included, suggest that this is likely due mainly to the presence of early-acting barriers. To test this hypothesis, we will soon be re-analyzing the genetic architecture of all of the previously-analyzed components of pre- and post-zygotic reproductive isolation [9-15] using the maps described in the current study. We will thus be able to test whether or not QTLs underlying the same phenotypes occur on the same or different linkage groups in *I. fulva *and *I. brevicaulis*. These QTLs (specifically the markers closely linked to those QTLs) will then serve as important testable hypotheses that will allow us to determine what specific regions of the genome (underlying which type of QTLs) are involved in introgression between *I. fulva *and *I. brevicaulis *in natural hybrid zones.

## Authors' contributions

ST developed the cDNA libraries, produced the ESTs, developed and screened the DNA markers, performed molecular and statistical genetic analyses, and assisted with drafting the manuscript. RAO assisted with the molecular analyses. SJK, MLA, and NHM designed and coordinated the study, and assisted with statistical analyses drafting the manuscript. All authors read and approved the final manuscript.

## Supplementary Material

Additional file 1 **Composite linkage map**. Composite genetic linkage map of *I. brevicaulis *and *I. fulva *based on 283 EST-SSR marker loci genotyped in 94 progeny of backcross mapping population BCIB, and 92 progeny of backcross mapping population BCIF. The genetic linkage groups were labeled from 1 to 21 in the order of their genetic map lengths in cM. Click here for file

Additional file 2 **EST genotyping data**. Polymorphisms and map locations of the 526 EST-SSR markers genotyped in the mapping parents IB25 and IF174. Click here for file
