# Supplementary figures and images for: Transmission ratio distortion results in asymmetric introgression in Louisiana Iris
Source: BMC Plant Biol. 2010 Mar 18;10:48. doi: 10.1186/1471-2229-10-48 (PMC2923522; doi:10.1186/1471-2229-10-48)

## Slide 1
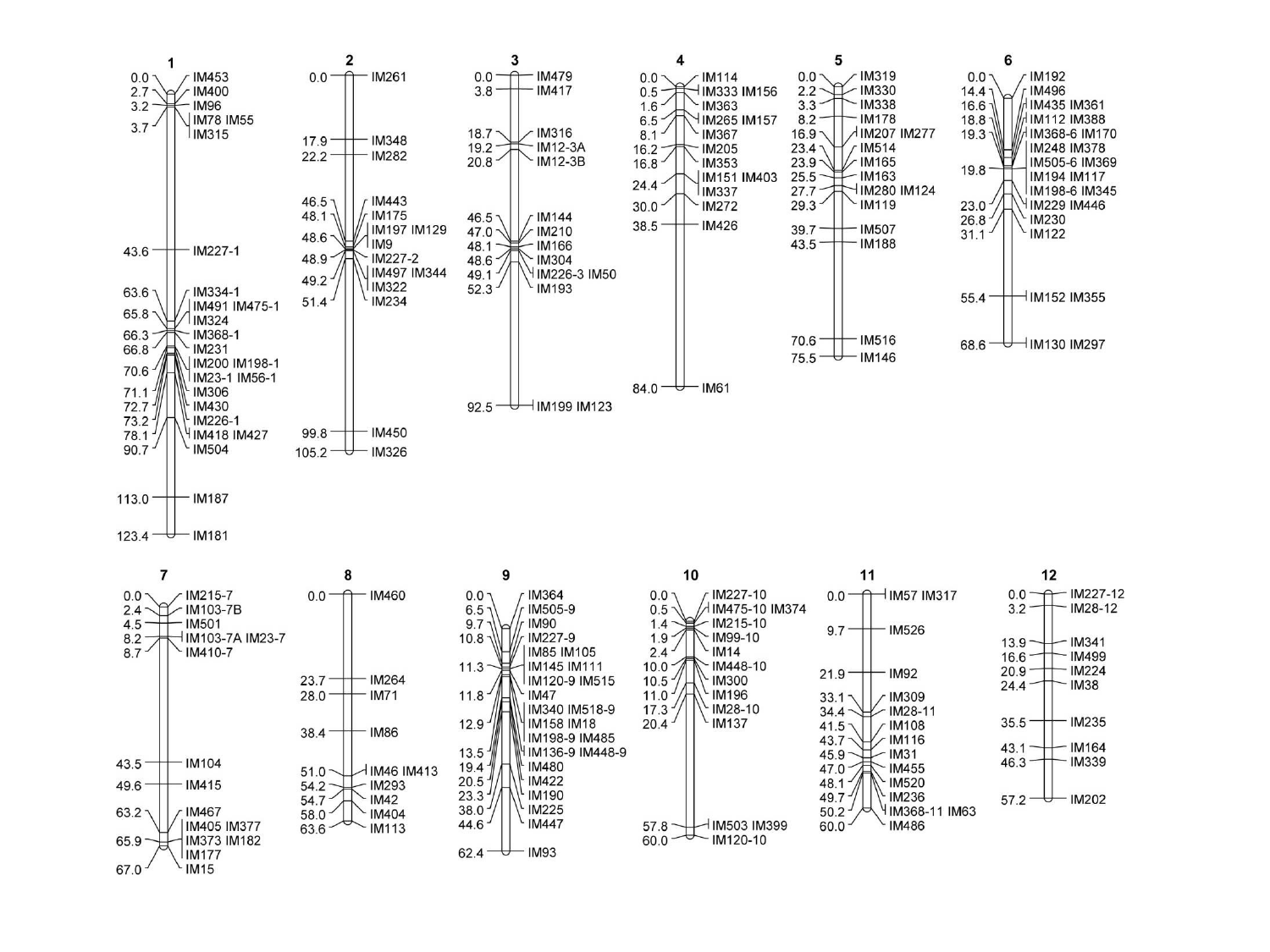

## Slide 2
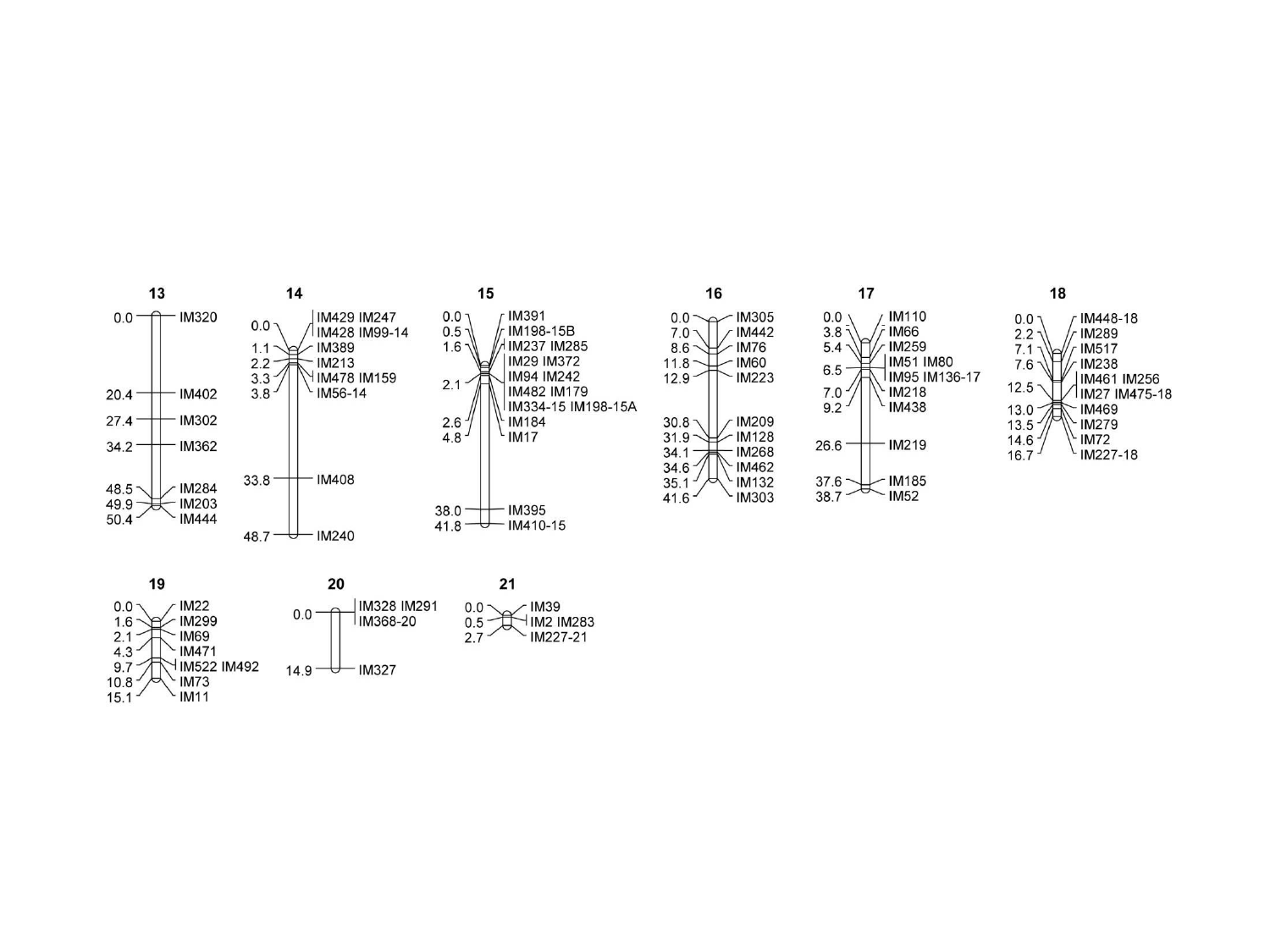

Supplement: Additional file 1 — Composite linkage map. Composite genetic linkage map of I. brevicaulis and I. fulva based on 283 EST-SSR marker loci genotyped in 94 progeny of backcross mapping population BCIB, and 92 progeny of backcross mapping population BCIF. The genetic linkage groups were labeled from 1 to 21 in the order of their genetic map lengths in cM. [file 1471-2229-10-48-S1.PPT]
